# Network pharmacology-based research on the effect of angelicin on osteosarcoma and the underlying mechanism

**DOI:** 10.18632/aging.204786

**Published:** 2023-06-10

**Authors:** Yafang Zhang, Junqiang Wei, Lingwei Kong, Mingze Song, Yange Zhang, Xiangyu Xiao, Haiying Cao, Zhehong Li, Ning Yang, Yu Jin

**Affiliations:** 1 Department of Traumatology and Orthopaedics, Affiliated Hospital of Chengde Medical University, Chengde 067000, Hebei, China; 2 Department of General Surgery, Beijing Shijitan Hospital, Capital Medical University, Beijing 100038, China; 3 Central Laboratory, Affiliated Hospital of Chengde Medical University, Chengde 067000, Hebei, China

**Keywords:** angelicin, osteosarcoma, network pharmacology, apoptosis, migration, proliferation

## Abstract

To explore the antitumor effects of angelicin on osteosarcoma and the underlying mechanism. We aimed to elucidate the mechanism by network pharmacology, molecular docking, and *in vitro* experiments. We analyzed a PPI network of potential angelicin targets in the treatment of osteosarcoma and identified hub targets. We systematically performed GO and KEGG enrichment analyses of the potential targets of angelicin, and we predicted it function in osteosarcoma treatment and the underlying molecular mechanism. Through molecular docking, the interactions between hub targets and angelicin were simulated, and then, the hub targets of angelicin were identified. Based on these results, we validated the effects of angelicin on osteosarcoma cells by conducting *in vitro* experiments. The PPI network analysis of potential therapeutic targets identified four apoptosis-related hub targets, namely, BCL-2, Casp9, BAX and BIRC 2. GO and KEGG enrichment analyses demonstrated that angelicin regulates osteosarcoma cell apoptosis. Molecular docking results indicated that angelicin can freely bind to the hub targets listed above. *In vitro* experiments showed that angelicin promoted osteosarcoma cell apoptosis in a dose-dependent manner and inhibited osteosarcoma cell migration and proliferation in a time- and dose-dependent manner. The RT-PCR results showed that angelicin simultaneously promoted the mRNA expression of Bcl-2 and Casp9 and inhibited the mRNA expression of BAX and BIRC 2. Angelicin promotes osteosarcoma cell apoptosis and inhibits osteosarcoma cell proliferation and migration by activating a signaling network that is composed of hub targets that link multiple signaling pathways. Angelicin could become an alternative drug for the treatment of osteosarcoma.

## INTRODUCTION

Osteosarcoma (OS) is the most common bone malignancy in children and adolescents [1, 2] and it accounts for 0.2% of all malignancy cases [3]. The current standard of care for OS includes neoadjuvant chemotherapy, radical resection and adjuvant chemotherapy [4]. Because of the use of these comprehensive treatments, the 5-year overall survival rate of primary patients OS has reached approximately 60–70% [4]. However, the 5-year overall survival rate decreases to lower than 15% after the occurrence of distant metastasis [4, 5]. Although new drugs for OS treatment, such as immune checkpoint inhibitors and targeted drugs [6, 7], are constantly being explored, the overall survival rate of OS has still not significantly improved in the past 30 years [8]. There is still an urgent need to discover new treatment options.

Angelicin is one of the main active ingredients of *Angelica* and *Angelica dahurica* [9]. Existing evidence has proven that angelicin exerts inhibitory effects on tumor cells by inhibiting proliferation, apoptosis, migration, and invasion [9–12]. However, research related to the role of Angelicin inhibiting OS and its underlying mechanism is insufficient.

Recently, network pharmacology has offered a new strategy for exploring the relationships between drugs and diseases by integrating systems biology, multidirectional pharmaceutical biology, bioinformatics, and computer science [13, 14]. Currently, there are no published studies on the effect of angelicin on the biological behavior of OS cells. In this study, we used network pharmacology and molecular docking techniques and *in vitro* experiments to explore whether angelicin affects the biological behavior of OS and its specific mechanism.

## MATERIALS AND METHODS

**Figure 1:**
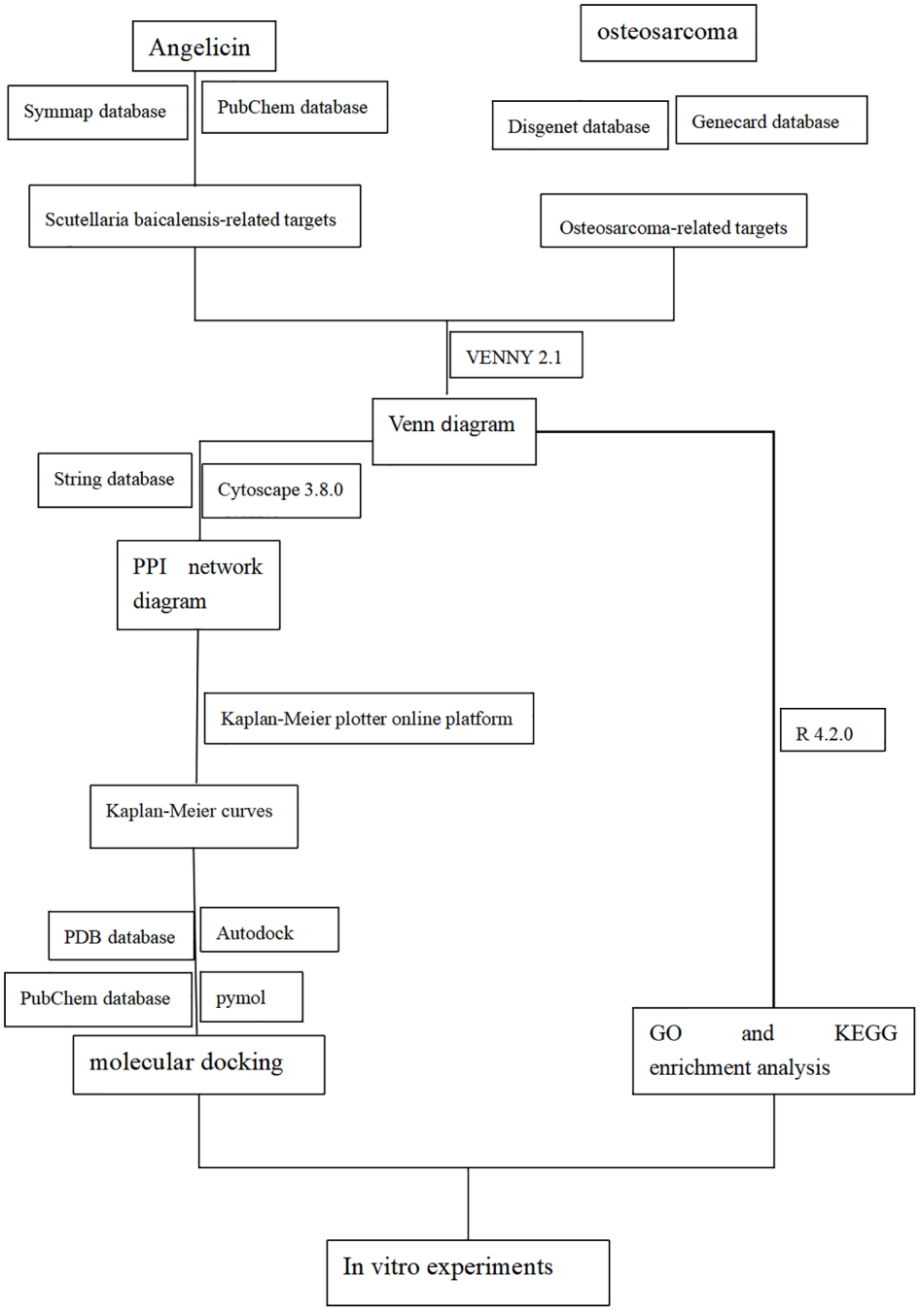
Flow diagram of the experimental design for studying the function of angelicin in the treatment of osteosarcoma.

The flow chart of the study design is shown in Figure 1.

### Identification of angelicin targets

All the active ingredients of Angelicin were obtained from the Pubchem (https://pubchem.ncbi.nlm.nih.gov/) and SymMap (http://www.symmap.org) databases with the keyword “Angelicin”. PubChem: that includes a substantial amount of data related to small organic molecule bioactivity and is funded by the National Institutes of Health (NIH). The SymMap database is a traditional Chinese medicine (TCM) evidence association database that includes an immeasurable amount of information about herbal medicines, their ingredients, and their drug targets; the TCM symptoms, Western medicine symptoms, and diseases associated with these symptoms that these medicines are used to treat; and correlations among these data. In this way, the SymMap database correlates TCM and Western medicine at levels ranging from the phenotypic level to the molecular level.

### Identification of OS targets

A search was performed in the DisGeNET database (https://www.disgenet.org/) and the GeneCards database (https://www.genecards.org/) using the keyword “Osteosarcoma” and using “*Homo sapiens*” as the filtering criterion. In addition, the targets obtained from the DisGeNET database with a score >0.8 were screened. The GeneCards database is a search platform that retrieves genes that are associated with human diseases from 150+ web sources. Genes were searched on the two platforms using the keyword “Osteosarcoma”. The retrieved data include information about Osteosarcoma, such as gene names and gene IDs. The targets that were obtained from these two databases were then integrated, and duplicates were removed, ultimately yielding genes related to “*Homo sapiens* OS”.

### Construction of a PPI network diagram

The identified angelicin-related targets and *Homo sapiens* OS-related targets were imported into an online Venn diagram mapping platform (https://bioinfogp.cnb.csic.es/tools/venny/). The region where the two circles of the Venn diagram intersect indicates the potential targets of angelicin in the treatment of OS. The targets that were related to the mechanism by which angelicin treats OS were imported into the STRING database. A PPI network diagram was generated with a confidence level of 0.7, and it was imported into the STRING app in Cytoscape software for further processing and analysis.

### Hub gene analysis

To further analyze the PPI network and identify hub targets, we imported the PPI network into Cytoscape 3.8.0 software and analyzed the network graph with Network Analyzer. Information on the degree, betweenness centrality (BC), and closeness centrality (CC) of the corresponding targets was obtained. We used degree≥degree median, CC≤CC median, and BC≥BC median as the screening criteria to identify the hub targets.

### Gene ontology (GO) and Kyoto Encyclopedia of Genes and Genomes (KEGG) analyses

To further analyze the potential mechanism underlying the function of angelicin in OS treatment, we enriched the targets mentioned above with the Bioconductor Cluster Profiler package in R software. According to the GO enrichment analysis, the potential therapeutic targets of angelicin in the treatment of OS are described in terms of biological processes, cellular components, and molecular functions. Additionally, the potential signaling pathways by which angelicin functions in the treatment of OS were explored by KEGG enrichment analysis.

### Molecular docking

To investigate the potential interaction between angelicin and its potential targets for the treatment of OS, we performed molecular docking between angelicin and the hub genes based on the principle of semiflexible molecular docking. The leading software for molecular docking is AutoDock, which includes AutoDock tools and AutoDock Vina. Angelicin and hub targets were processed with AutoDock tools, and the docking center of the hub targets was determined. The 3D structures of the target proteins were obtained from the PDB online platform, and the 3D structures of angelicin were obtained from the PubChem online platform.

### Cell culture

The human OS cell line MG63 (Procell CL-0157) was kindly provided by Procell Life Science and Technology Co., Ltd. MG63 Cells were grown in 90% modified Eagle’s medium (MEM) supplemented with 10% fetal bovine serum (FBS), 100 U/mL penicillin, and 100 μmol/L streptomycin, and incubated at 37°C in a humidified atmosphere of 5% CO2.

### Cell counting Kit-8 (CCK8) assay

MG63 cells were treated with trypsin, grown in 96-well plates (3000 cells/well), and cultured with media supplemented with various concentrations of angelicin. The cells were incubated in a 37°C cell culture incubator for 24 h, 48 h or 72 h. At each time point, the plates were removed, the medium in the corresponding wells was gently aspirated, and the cells were gently washed with sterile PBS. CCK8 and serum-free medium were mixed at a ratio of 1:9, and 100 μl of the mixture was slowly added to each well. The cells were incubated in a cell culture chamber at 37°C for 1 h. The OD value of each well was measured at 450 nm by a multifunctional enzyme marker. Cell activity (%) = (OD value of treatment group-OD value of blank control group)/(OD value of experimental control group-OD value of blank control group) × 100%.

### Flow cytometry

According to the instructions, we analyzed the effect of angelicin on the apoptosis of MG63 cells by flow cytometry. MG63 cells were seeded in 6-well plates at a density of 5 × 10^5^ cells per well; when the cells had completely adhered to the well, the original medium was discarded, and the cells were washed 2 times with PBS. Angelicin was added at different concentrations and incubated for 24 h. The cells were stained with propidium iodide (PI) and annexin V-fluorescein isothiocyanate (FITC). ACEA NovoCyteTM (Biosciences, San Diego, CA, USA) equipment was used to perform the flow cytometry analysis. FlowJo (Version 10.0, Stanford, CA, USA) software was used to analyze the results.

### Wound healing experiment

MG63 OS cells in the logarithmic phase of growth were seeded in 6-well plates at a density of 3 × 10^5^ cells per well. Then, the cell culture plates were gently shaken to uniformly distribute the cells to form a monolayer, and the cells were prepared for scoring when they reached 95% confluence. A 200 μL pipette tip was used to form a vertical scratch along a sterile straightedge. Then, the treated wells were gently washed 3 times with PBS to remove the nonadherent cells, and the plates were imaged and photographed (0 h). Media supplemented with different concentrations of angelicin (50 and 200 μmol/L) were added, and the control group was incubated in the same volume of MEM supplemented with DMSO; the cells were cultured in an incubator at a constant temperature of 37°C with 5% CO_2_ for 24 h or 48 h, and then, the cells were photographed. The scratch area S was obtained using the scratch area analysis module of ImageJ software, and the scratch healing rate was calculated according to the following formula: wound healing rate = (1-(S24h/S0h)) × 100%.

### Reverse transcription (RT) PCR technology

Total RNA was extracted from cells using an RNA extraction kit, first-strand complementary DNA (cDNA) was synthesized from the total RNA using an RNA reverse transcriptase kit, and quantitative real-time reverse transcription polymerase chain reaction (qRT-PCR) was performed using SYBR Green reagent (TaKaRa, Japan) according to the instructions. The primer sequences are shown in Table 1.

**Table 1:** The primer sequences of Bax, Bcl-2, CASP9, BIRC2.

**Primer name**	**Primer sequences (5′ to 3′)**
Bcl2 Forward	CAGGATAACGGAGGCTGGGATG
Bcl2 Reverse	AGAAATCAAACAGAGGCCGCA
Bax Forward	CCCGAGAGGTCTTTTTCCGAG
Bax Reverse	CCAGCCCATGATGGTTCTGAT
CASP9 Forward	CGCTAATGCTGTTTCGGT
CASP9 Reverse	AAGATAAGGCAGGGTGAGG
BIRC2 Forward	AGTGGTTTCCAAGGTGTGAGT
BIRC2 Reverse	AGCCCATTTCCAAGGCAGAT

### Statistical analysis

Statistical analysis of the bioinformatics data and mapping was performed by R ×64 4.0.3, Cytoscape 3.8.0, and PyMOL software. Statistical analysis of the *in vitro* experimental data was conducted using GraphPad Prism 9. The difference between two groups was assessed using one-way ANOVA. The statistical significance of the CCK8 results was assessed using two-way ANOVA. The data are presented as the means ± standard errors of the means (SEM), and a *p* value < 0.05 indicates statistical significance.

### Data availability statement

The original contributions presented in the study are included in the article Material, further inquiries can be directed to the corresponding authors.

## RESULTS

### Targets of angelicin

Using “angelicin” as the keyword, we screened its targets in the PubChem and SymMap databases. We identified 36 targets of angelicin by integrating and deweighting the screened targets, as shown in Table 2.

**Table 2:** Information about 36 angelicin targets.

**Gene symbol**	**Gene name**	**Gene symbol**	**Gene name**
BCL2	BCL2, Apoptosis Regulator	ABCB11	ATP-binding cassette subfamily B member 11
CASP3	Caspase 3	ABCB5	ATP-binding cassette sub-family B member 5
CYP1A1	Cytochrome P450 family 1 subfamily A member 1	ABCC1	Multidrug resistance-associated protein 1
ICAM1	Intercellular adhesion molecule 1	ABCC2	Multidrug resistance-associated protein 2
BAX	Apoptosis regulator BAX	ABCC3	Multidrug resistance-associated protein 3
BIRC2	Baculoviral IAP Repeat Containing 2	ABCC4	Multidrug resistance-associated protein 4
CASP9	Caspase 9	ACHE	Acetylcholinesterase
FAM102A	Protein FAM102A	AHR	Aryl hydrocarbon receptor
CYP1A1	Cytochrome P450 1A1	NR1H4	Bile acid receptor
CYP1A2	Cytochrome P450 1A2	SLC22A1	Solute carrier family 22 member 1
CYP2E1	Cytochrome P450 2E1	SLC22A2	Solute carrier family 22 member 2
CYP3A11	Cytochrome P450 3A11	SLC22A4	Solute carrier family 22 member 4
CYP7A1	Cytochrome P450 7A1	SLC22A5	Solute carrier family 22 member 5
FABP2	Fatty acid-binding protein 2	SLC22A6	Solute carrier family 22 member 6
GPT	Alanine aminotransferase 1	SLC22A8	Solute carrier family 22 member 8
SOD1	Superoxide dismutase (Cu-Zn)	SLC2A9	Solute carrier family 2, facilitated glucose transporter member 9
SULT2A1	Sulfotransferase 2A1		

### Targets of OS

The keyword “osteosarcoma” was used to screen the DisGeNET and GeneCards databases. A total of 4389 relevant targets were identified after integration and removal of duplicates.

### Constructing and analyzing the PPI network

**Figure 2:**
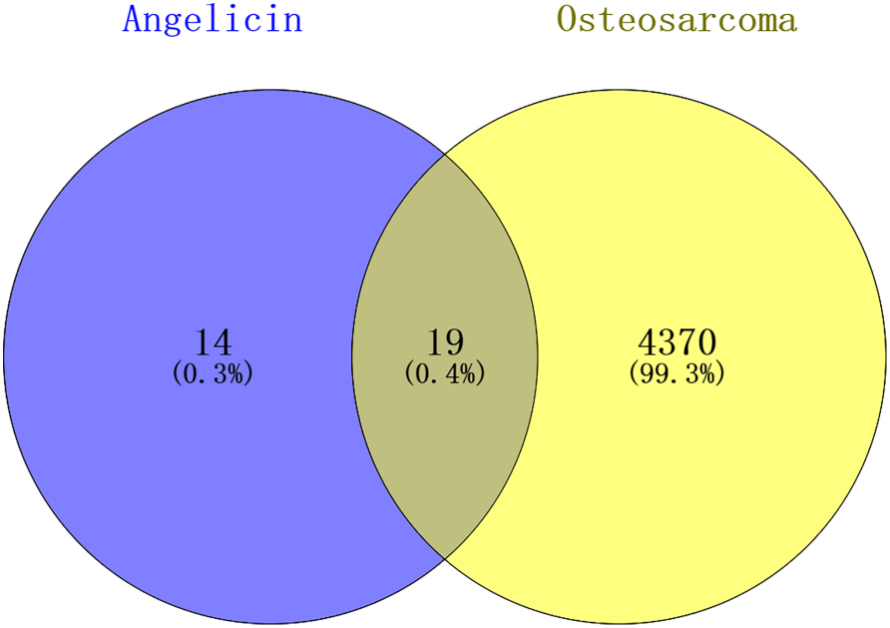
**Venn diagram showing the intersection of angelicin-related genes and osteosarcoma-related genes.** The angelicin-related targets are shown in the blue circle, and the osteosarcoma-related targets are shown in the yellow circle. The intersection of the two circles indicates potential targets of angelicin in osteosarcoma treatment.

**Figure 3:**
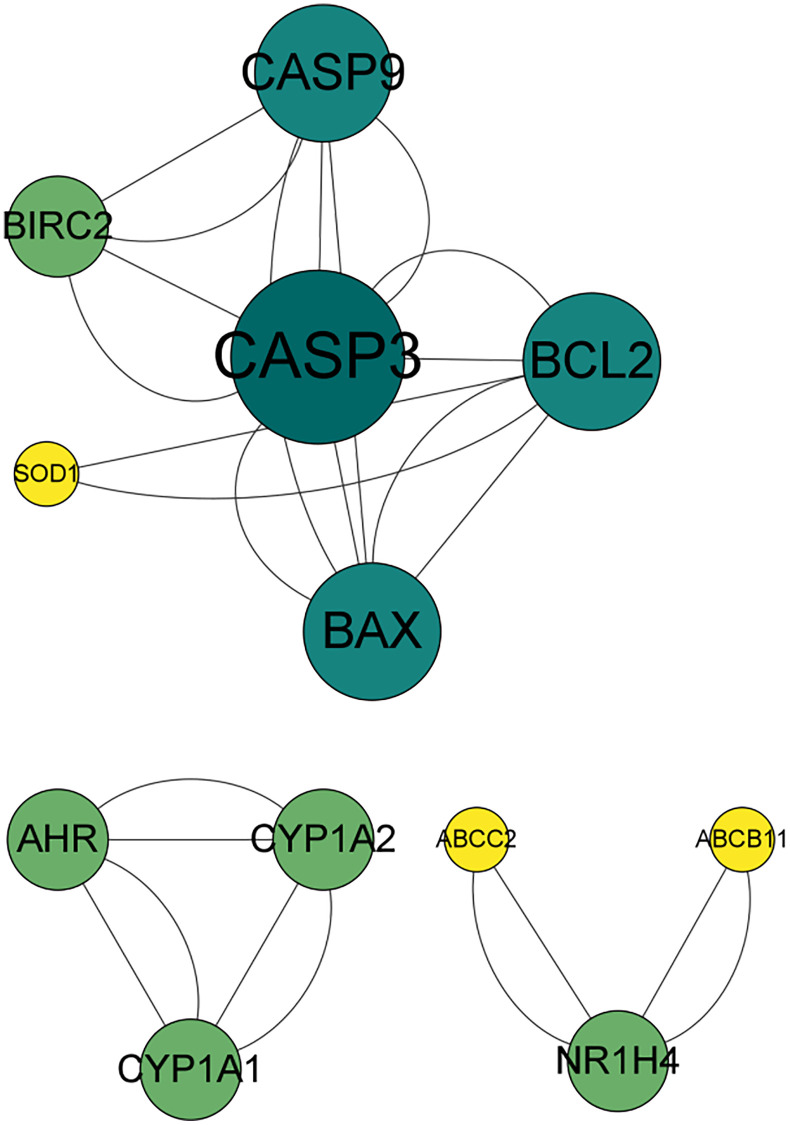
**PPI network of the effects of angelicin in the treatment of osteosarcoma.** The nodes represent potential therapeutic targets of angelicin in the treatment of osteosarcoma. The larger the node, the darker the color, the higher the target degree, and the higher the number of connections with other nodes.

Angelicin targets and OS therapeutic targets are shown in a Venn diagram (Figure 2). In total, 19 potential targets of angelicin in the treatment of OS were identified (Supplementary Table 1). The potential therapeutic targets were imported into the STRING online platform to generate PPI network diagrams, which were imported into Cytoscape software for processing and analysis (Figure 3). We determined the correlation coefficients of the PPI network, including degree, closeness centrality, and betweenness centrality (Supplementary Table 2). We used the screening criteria of degree≥degree median, CC≤CC median, and BC≥BC median to identify hub targets and showed median degree = 2, median CC = 0.714286, and median BC = 0. The hub targets, including the apoptosis regulator BAX (BAX), b-cell lymphoma-2 (Bcl-2), baculoviral IAP repeat-containing protein 2 (BIRC 2), and caspase 9 (Casp9), were identified based on degree≥2, CC≤0.714286, and BC≥0.

### GO and KEGG analysis

**Figure 4:**
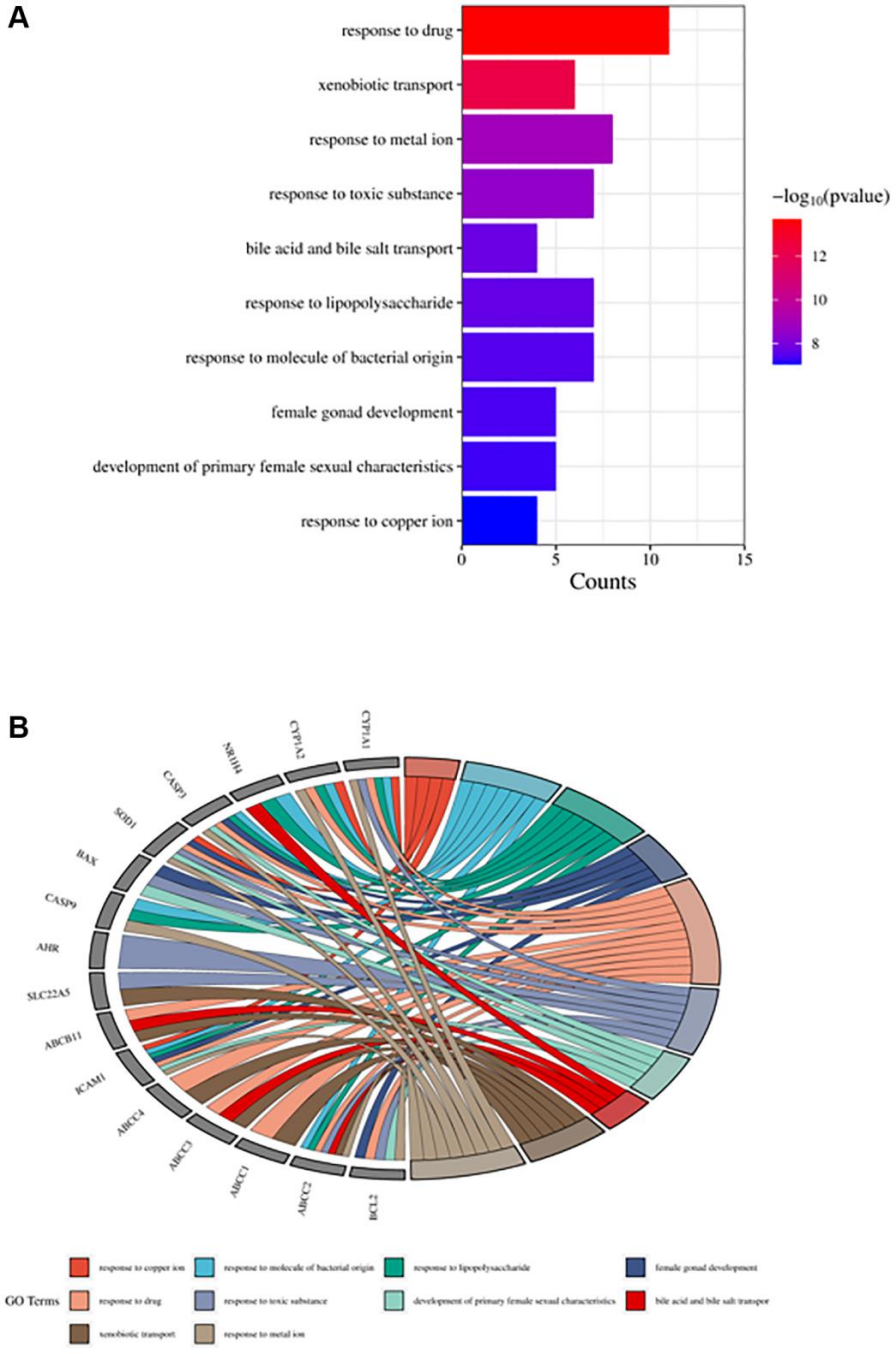
**Top ten significant biological process (BP) entries.** (**A**) GO enrichment analysis of therapeutic targets for biological processes. (**B**) Relationship between the therapeutic targets and biological processes.

**Figure 5:**
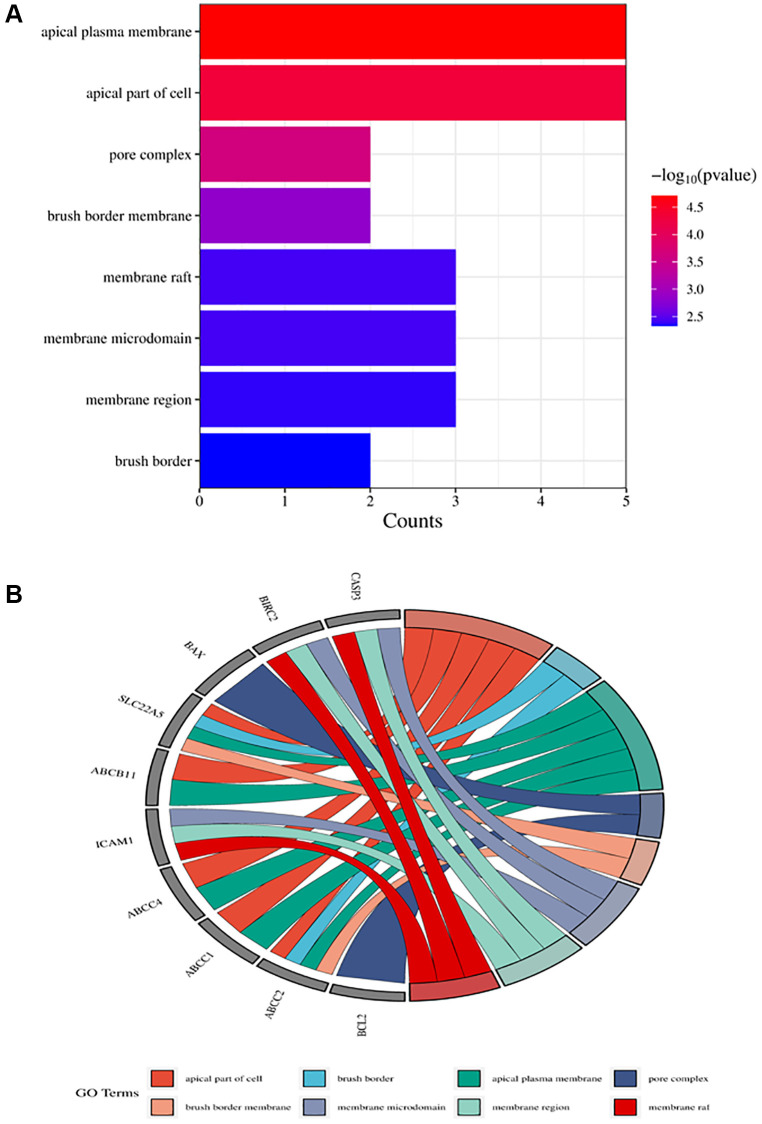
**Top ten significant cell component (CC) entries.** (**A**) GO enrichment analysis of therapeutic targets for cell component. (**B**) Relationship between the therapeutic targets and cell components.

**Figure 6:**
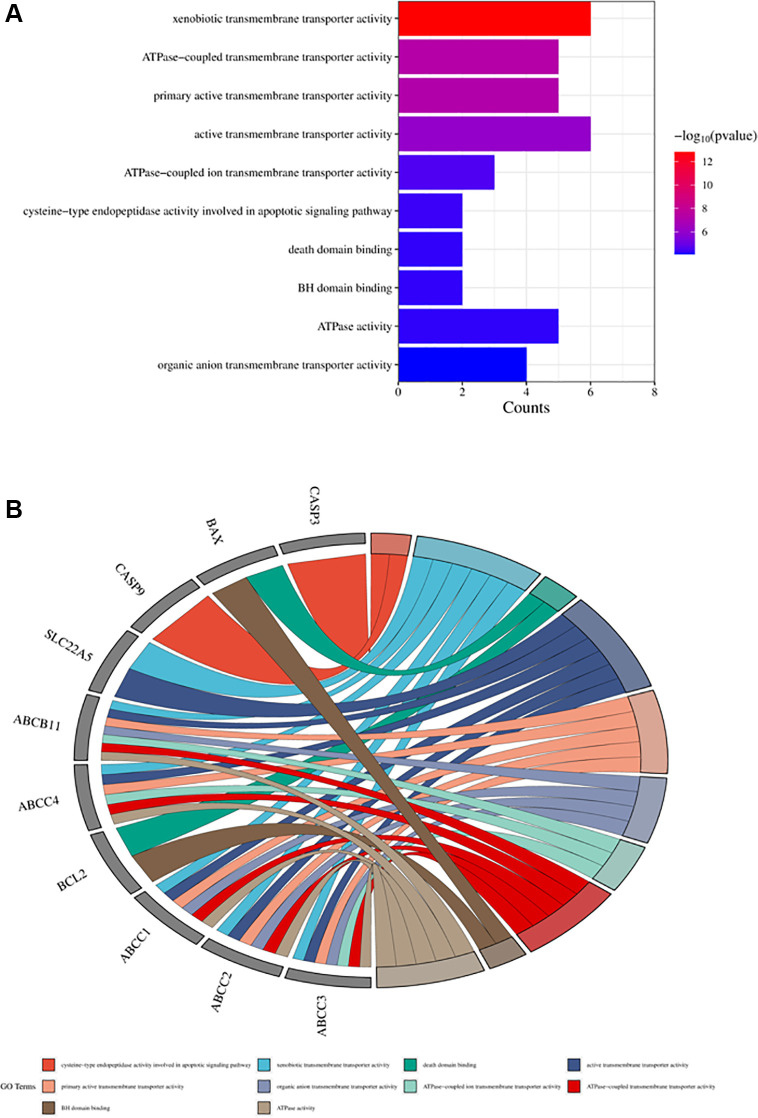
**Top ten significant molecular function (MF) entries.** (**A**) GO enrichment analysis of therapeutic targets for molecular function. (**B**) Relationship between the therapeutic targets and molecular functions.

**Figure 7:**
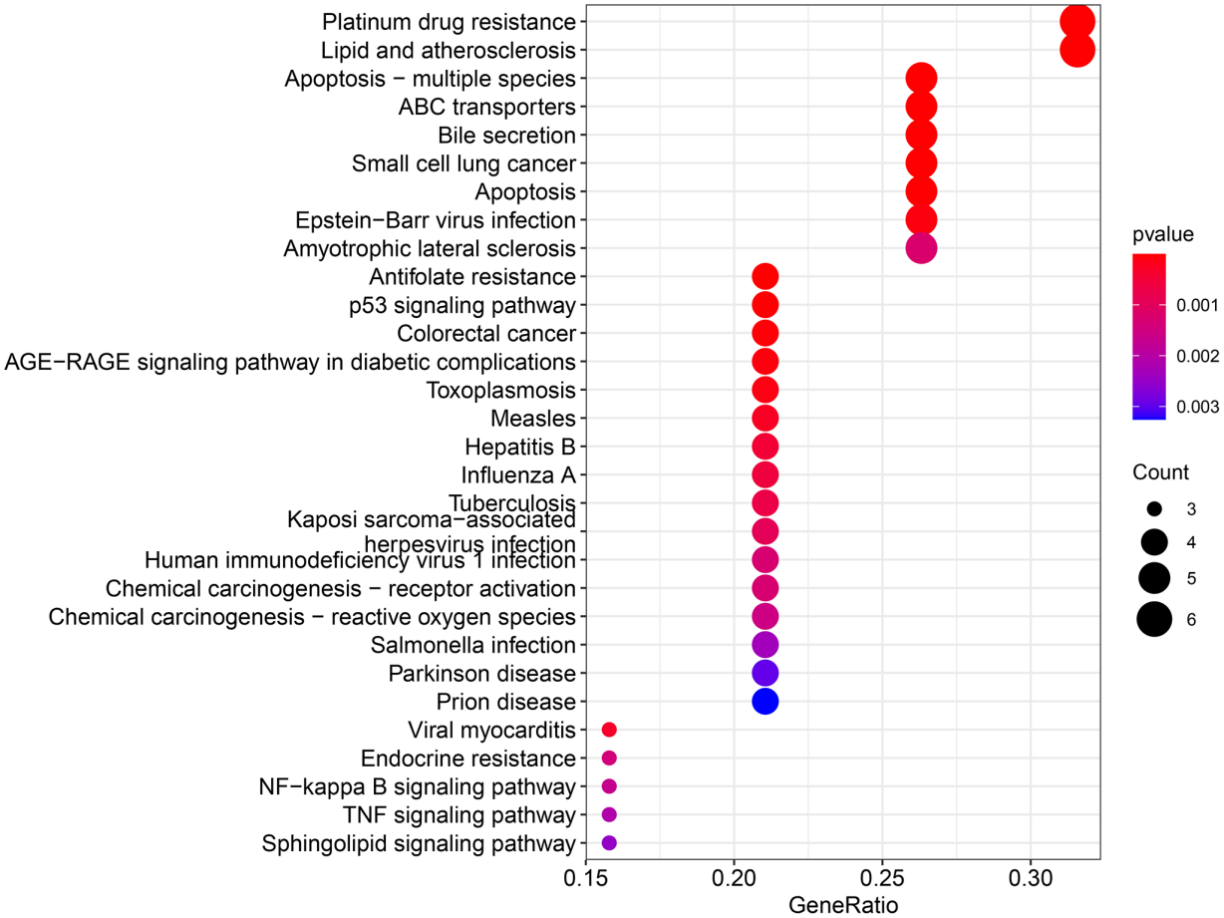
**KEGG enrichment analysis for therapeutic targets.** The larger the bubble, the more targets are enriched in that pathway, and the redder the color; the smaller the *P* value, the more meaningful the corresponding signaling pathway.

The obtained potential targets of angelicin in the treatment of OS were subjected to GO and KEGG enrichment analyses. The results of biological processes, cellular components, and molecular functions are shown in Supplementary Table 3. The top 10 biological processes and molecular functions and all the cellular components are shown in Figures 4–6; the relationships of the top 10 biological processes and cellular components and all the molecular functions with the potential targets are shown in Figures 4–6, respectively. The potential targets of angelicin in the treatment of OS are shown in Supplementary Table 4, and they were analyzed by KEGG enrichment analysis; the results showed that angelicin impacts osteosarcoma through multiple signaling pathways, such as apoptosis, the p53-related signaling pathway, the NF-kappa B signaling pathway, and the TNF signaling pathway. The top 30 signaling pathways are shown in Figure 7.

### Molecular docking simulation

**Figure 8:**
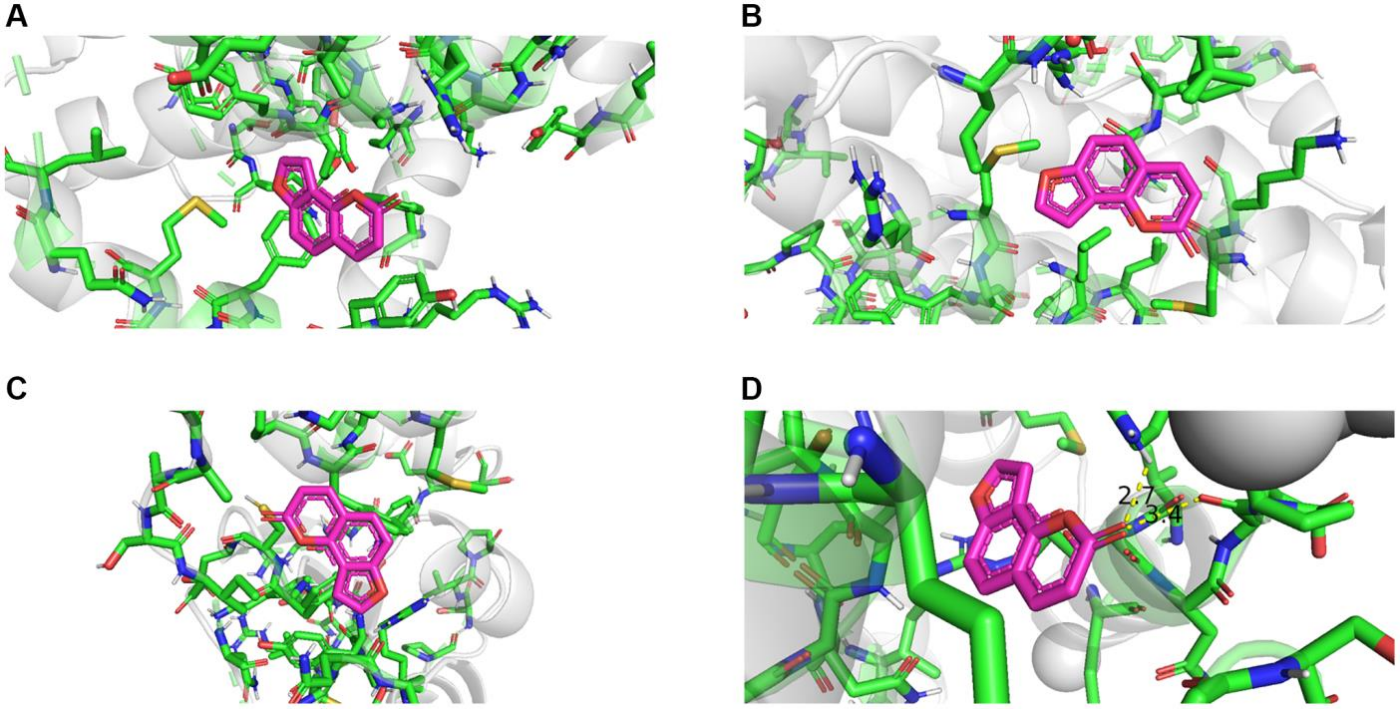
**Molecular docking results of angelicin and hub genes.** (**A**) Bcl 2 free energy: −6.2 kcal/mol. (**B**) BAX free energy: −5.7 kcal/mol. (**C**) BIRC 2 free energy: −5.3 kcal/mol. (**D**) Casp9 free energy: -5.8 kcal/mol).

Molecular docking of the identified hub genes was performed using AutoDock software. The molecular docking results were visualized using PyMOL software, and the docking results of the four hub targets are shown in Figure 8. The specific details of the docking results are shown in Supplementary Table 4. A binding free energy<−4 kcal/mol when one of the targets was docked with the small molecule of angelicin indicated that the target could freely bind to angelicin. According to molecular docking results, angelicin could bind freely to the hub targets, and the hub targets BAX, BRIC2, BCL2, and Casp9 may be effective targets by which angelicin affects osteosarcoma cells.

### Angelicin inhibits the proliferation of MG63 OS cells *in vitro*

**Figure 9:**
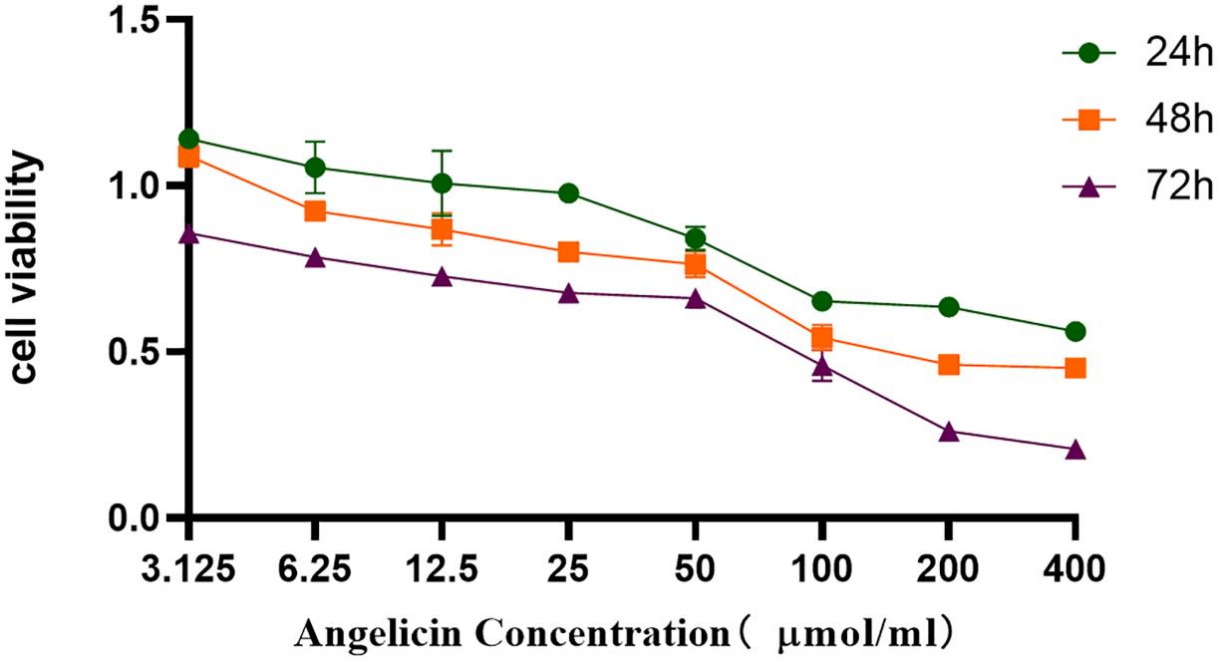
**CCK8 assay.** Angelicin inhibited the proliferation of osteosarcoma MG63 cells. The viability of osteosarcoma MG63 cells was measured at different time points (24 h, 48 h, or 72 h) of culture with media containing different angelicin concentrations (3.125–400 μmol/ml), and the results are shown as the mean ± standard deviation.

To investigate the effect of angelicin on the proliferation of OS cells, we used a CCK8 assay to determine MG63 cell viability after treatment with different concentrations of angelicin. The differences in cell viability among the 24 h, 48 h and 72 h groups were statistically significant (*P* < 0.0001). The differences in cell viability between the 50 μmol/L and 200 μmol/L groups were statistically significant (*P* < 0.0001) (Figure 9). The viability of MG63 cells gradually decreased as the angelicin concentration increased and the culture time increased. These results indicated that angelicin inhibited the proliferation of MG63 cells in a dose-dependent and time-dependent manner.

### Angelicin induces the apoptosis of MG63 OS cells *in vitro*

**Figure 10:**
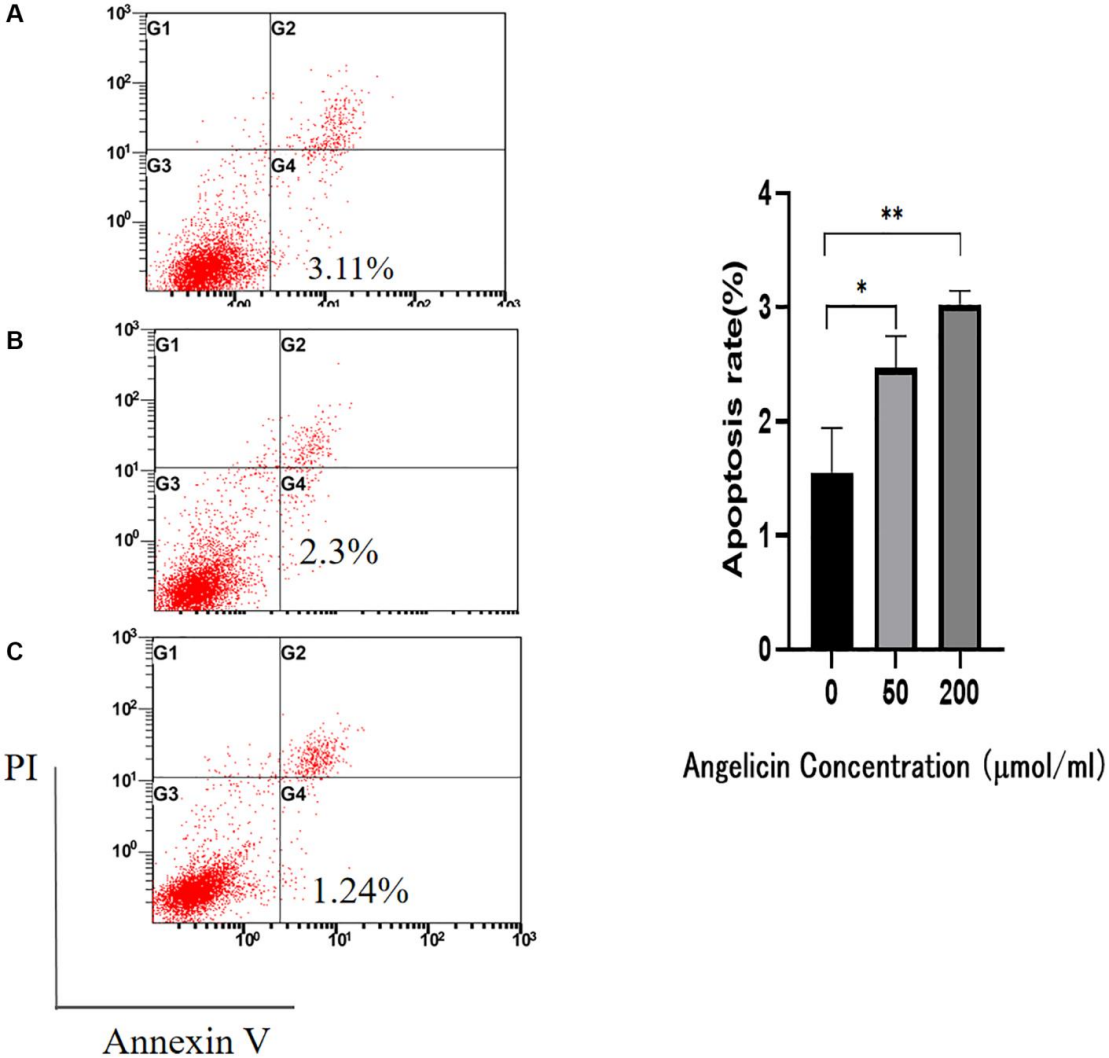
**Flow cytometry.** Angelicin promotes apoptosis in osteosarcoma MG63 cells. The apoptosis rate of osteosarcoma MG63 cells was measured after 24 h of culture with media containing different concentrations of angelicin (**A**) 0 μmol/ml, (**B**) 50 μmol/ml, and (**C**) 200 μmol/ml). The results of quantitative analysis are shown as the mean ± S.D. of three independent experiments. ^*^Significant difference compared to the control group: *p* < 0.05, ^**^p < 0.01.

We used flow cytometry to analyze the apoptosis of MG63 cells after treatment with different concentrations of angelicin. The apoptosis rates of the angelicin-treated groups were statistically significantly different compared with that of the control group (*P* < 0.05). The apoptosis rate of OS MG63 cells gradually increased with increasing angelicin concentrations (Figure 10). These results suggest that angelicin promotes the apoptosis of OS MG63 cells in a dose-dependent manner.

### Angelicin inhibits the migration of OS MG63 cells *in vitro*

**Figure 11:**
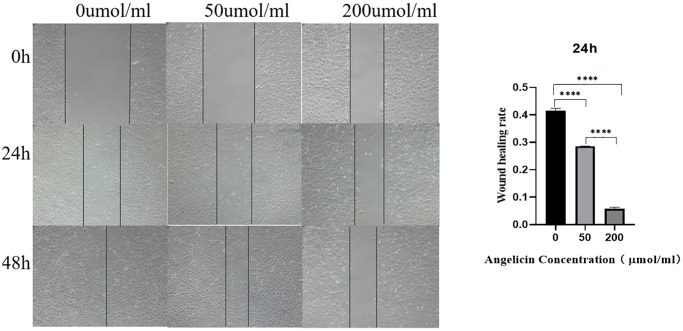
**Wound healing experiment.** Angelicin inhibits the migration of osteosarcoma MG63 cells. MG63 osteosarcoma cell wound healing results at different time points (0 h, 24 h, or 48 h) of culture with media containing different angelicin concentrations (0 μmol/ml, 50 μmol, or 200 μmol/ml). The results of quantitative analysis are shown as the mean ± S.D. of three independent experiments. ^*^Significant difference between the two groups: *p* < 0.05, ^****^p < 0.0001.

We performed the wound healing assay to evaluate the migration rate of MG63 cells, and the results showed that the differences in the wound healing rate among the different concentration groups were statistically significant (*P* < 0.05). The wound healing rate of OS cells gradually decreased with increasing angelicin concentration (Figure 11), which indicates that angelicin inhibited the migration of OS MG63 cells in a dose-dependent manner.

### RT-PCR experiments

**Figure 12:**
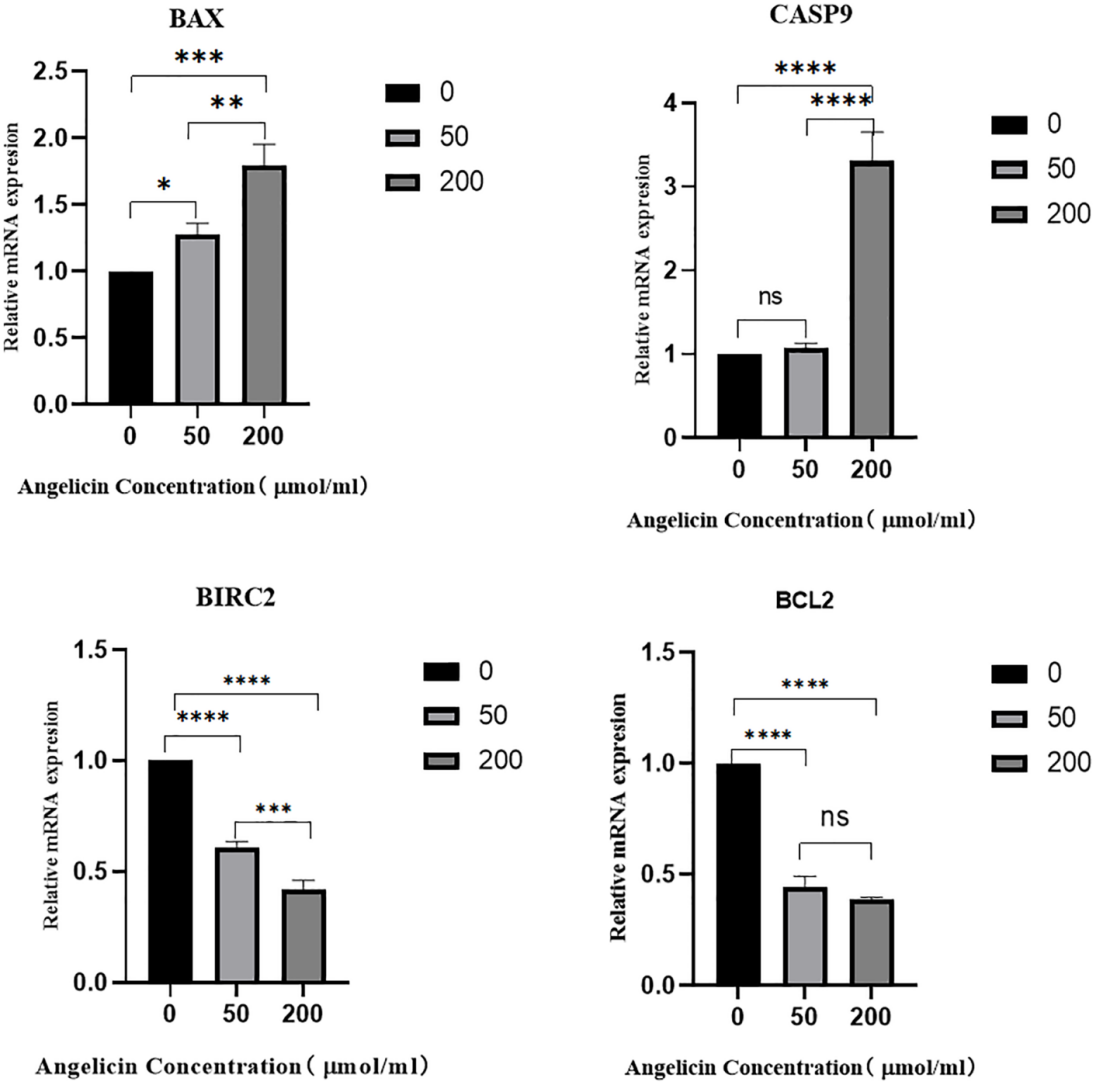
**The effect of angelicin on the expression of hub target genes in osteosarcoma MG63 cells.** The mRNA expression of the hub genes in osteosarcoma MG63 cells was measured after 24 h of culture with media containing different concentrations (0 μmol/ml, 50 μmol/ml, or 200 μmol/ml) of angelicin; (**A**) BAX, (**B**) Casp9, (**C**) BIRC2, (**D**) Bcl2; ns (No significance): No statistically significant difference between the two groups. When *P* < 0.05, ^*^the difference between the two groups is statistically significant. ^*^P < 0.05, ^**^P < 0.01, ^***^P < 0.001, ^****^P < 0.0001.

To measure the mRNA expression of pivotal targets in OS MG63 cells after treatment with different concentrations of angelicin, we performed RT-PCR experiments, and the results demonstrated that the expression of BAX and Casp9 gradually increased, while the expression of the antiapoptotic proteins Bcl-2 and BIRC2 gradually decreased with increasing angelicin concentration. The differences in mRNA expression among the different concentration groups were statistically significant (*P* < 0.05), and the results are shown in Figure 12.

## DISCUSSION

Osteosarcoma is the most common malignant tumor, accounting for approximately 55% of all bone tumors [15]. OS is more common in children and adolescents and poses great risks to human health [1, 16]. The current clinical treatment is so-called “sandwich therapy”, which consists of neoadjuvant chemotherapy, radical resection surgery and adjuvant chemotherapy [4, 17]. The 5-year overall survival rate of primary OS patients who receive classical treatment is approximately 60–70% [6]; however, OS has a high probability of local recurrence and distant metastasis [18]. Approximately 15–20% of OS patients have distant metastases when they are first diagnosed [19], and pulmonary metastasis is the most common metastatic event [20]. The 5-year survival rate decreases to less than 15% once distant metastasis occurs [3]. Although new treatments and pathological mechanisms are being explored, no significant progress has been made over the past several decades [6]. In this research, we showed that angelicin exerts an inhibitory effect on osteosarcoma cells by suppressing proliferation and migration and promoting apoptosis. Angelicin is one of the furanocoumarins found in many Chinese herbal medicines, such as *Angelica archangelica* and Paeoniae Radix Alba [9, 21]. Existing studies have shown that angelicin has antitumor effects on many types of malignancies, such as liver cancer and lung cancer [9, 10]. Inspired by the above research, we first investigated the role of angelicin in OS.

In this study, we intersected angelicin therapeutic targets and OS-related targets that were identified in several databases and found potential therapeutic targets through which angelicin functions in the treatment of OS. Furthermore, we found the molecular hub targets of angelicin. Then, we screened the hub targets, including Casp9, BIRC2, BAX, and BCL2, of which BCL2 and Casp9 are proapoptotic proteins, and BAX and BIRC2 are antiapoptotic proteins [22]. All four hub target genes are closely associated with apoptosis. Moreover, the GO and KEGG enrichment results showed that the potential targets of angelicin in the treatment of OS were enriched in multiple signaling pathways. In addition, we found that the hub targets are involved in the transduction of multiple signaling pathways. For example, BAX is involved in the signaling of apoptosis, apoptosis-multiple species, and the p53 signaling pathway. BCL2 is involved in signaling pathways such as apoptosis, apoptosis-multiple species, the p53 signaling pathway, and the NF-κB signaling pathway. BIRC2 is involved in apoptosis, apoptosis-multiple species, the NF-κB signaling pathway, and the TNF signaling pathway. Numerous studies have demonstrated that multiple signaling pathways are involved in regulating the multiple biological behaviors of osteosarcoma cells [23–27]. The hub targets connect multiple signaling pathways to form an interactive signaling network, through which *Angelica* affects the biological behaviors of osteosarcoma. When the NF-κB signaling pathway is inhibited, the biological behaviors of osteosarcoma, such as proliferation, migration, and apoptosis, are suppressed [25, 28, 29]. When p53 is activated, it inhibits the proliferation and migration of osteosarcoma cells and promotes the apoptosis of osteosarcoma cells [27, 30, 31]. We found that angelicin can inhibit the proliferation and migration of osteosarcoma by modulating the signaling network formed by connections of hub targets. Based on these results, we further explored the effect of angelicin on OS. Since the hub targets are all apoptosis-related proteins and are enriched in several apoptosis-related signaling pathways according to the KEGG results, we focused on verifying the effect of angelicin on the apoptosis of osteosarcoma cells. The results showed that angelicin promotes OS cell apoptosis in a dose-dependent manner. The *in vitro* results further confirmed the network pharmacological results. It was also demonstrated that angelicin inhibits osteosarcoma cell proliferation and migration in a dose-dependent manner and promotes osteosarcoma cell apoptosis in a dose-dependent manner.

The network pharmacology, molecular docking, and *in vitro* experiments described above show that angelicin inhibits OS cell proliferation and migration and promotes OS cell apoptosis upregulating Casp9 and Bcl-2 gene expression and downregulating BAX and BIRC2 gene expression, thereby regulating apoptosis in multiple species, apoptosis, the P53 signaling pathway, the NF-κB signaling pathway, and the TNF signaling pathway to form a signaling pathway network.

Through pharmacological and molecular docking assays, we discovered the regulatory effect of angelicin on osteosarcoma cells. Based on these results, we verified with *in vitro* experiments that angelicin inhibits osteosarcoma cell proliferation and migration and promotes osteosarcoma cell apoptosis. We are the first to observe these results. Network pharmacology is an efficient tool that is used for the investigation of the mechanisms of action of drugs. However, it is clear that the present study still has some limitations. This study did not examine the specific signaling pathways that are regulated by angelicin in osteosarcoma cells, but we have predicted which pathways are targeted by angelicin via network pharmacology; molecular biological validation is our next step. In addition, *in vivo* experiments were not conducted in this study, and the inhibitory effect of angelicin on osteosarcoma *in vivo* was not explored; we will pursue these experiments in future work. Third, the exploration of the loci of apoptotic proteins on which angelicin acts is another focus of our future work.

## CONCLUSION

We found that via the regulation of multiple signaling pathways, angelicin inhibited osteosarcoma cell proliferation in a time- and dose-dependent manner, inhibited osteosarcoma cell migration in a dose-dependent manner, and promoted osteosarcoma cell apoptosis in a dose-dependent manner. Angelicin could be an alternative medicine for the treatment of osteosarcoma, and it still needs to be explored in depth.

## Supplementary Materials

Supplementary Tables 1-2 and 4

Supplementary Table 3
