# Supplementary material for: Network pharmacology-based research on the effect of angelicin on osteosarcoma and the underlying mechanism
Source: Aging (Albany NY). 2023 Jun 10;15(11):5125–43. doi: 10.18632/aging.204786 (PMC10292874; doi:10.18632/aging.204786)
Supplement: Supplementary Tables 1-2 and 4 [file aging-15-204786-s001.pdf]

SUPPLEMENTARY TABLES

Supplementary Table 1. Information about 19 potential targets of angelicin in the treatment of osteosarcoma.

Gene symbol	Gene name	Gene symbol	Gene name
BCL2	Apoptosis regulator Bcl-2	ABCC3	ATP-binding cassette sub-family C member 3
CASP3	Caspase-3	ABCC4	ATP-binding cassette sub-family C member 4
CYP1A1	Cytochrome P450 1A	ACHE	Acetylcholinesterase
ICAM1	Inter-cellular adhesion molecule 1	AHR	Aryl hydrocarbon receptor
BAX	Apoptosis regulator BAX	CYP1A2	Cytochrome P450 1A2
BIRC2	Baculoviral IAP repeat-containing protein 2	GPT	Alanine aminotransferase 1
CASP9	Caspase-9	NR1H4	Bile acid receptor
ABCB11	ATP-binding cassette sub-family C member 11	SLC22A5	Solute carrier family 22 member 5
ABCC1	ATP-binding cassette sub-family C member 1	SOD1	Superoxide dismutase (Cu-Zn)
ABCC2	ATP-binding cassette sub-family C member 2		

Supplementary Table 2. Corresponding degree, closeness centrality, and betweenness centrality of potential targets of angelicin in the treatment of osteosarcoma in the PPI network.

Gene symbol	Degree	Closeness centrality	Betweenness centrality
SOD1	4	0.428571429	0.014705882
ABCC1	3	0.461538462	0.07762216
BAX	3	0.391304348	0
BCL2	4	0.4	0.002178649
CYP1A2	5	0.486486486	0.059508248
ACHE	2	0.461538462	0
ICAM1	2	0.461538462	0
BIRC2	2	0.382978723	0
ABCC4	2	0.391304348	0.006053533
ABCC3	3	0.409090909	0.011500156
AHR	3	0.375	0.002396514
ABCB11	5	0.5	0.033769063
CYP1A1	4	0.4	0.00681606
CASP9	6	0.514285714	0.115328354
NR1H4	7	0.529411765	0.174883287
SLC22A5	4	0.461538462	0.087192655
GPT	8	0.642857143	0.414114535
ABCC2	6	0.529411765	0.080003112
CASP3	9	0.580645161	0.260333022

Supplementary Table 4. KEGG enrichment results for potential targets of angelicin in the treatment of osteosarcoma.

Description	*p* value	Description	*p* value
Apoptosis - multiple species	7.69E-09	Human immunodeficiency virus 1 infection	0.001289001
Platinum drug resistance	1.06E-08	Chemical carcinogenesis - receptor activation	0.001289001
ABC transporters	4.58E-08	Endocrine resistance	0.00143819
Antifolate resistance	5.66E-07	Chemical carcinogenesis - reactive oxygen species	0.001554578
Bile secretion	1.46E-06	NF-kappa B signaling pathway	0.001706717
Small cell lung cancer	1.72E-06	TNF signaling pathway	0.002110974
Lipid and atherosclerosis	6.56E-06	Salmonella infection	0.0023311
Apoptosis	1.18E-05	Sphingolipid signaling pathway	0.002509952
p53 signaling pathway	2.11E-05	Parkinson disease	0.002964368
Colorectal cancer	4.04E-05	Prion disease	0.003256603
AGE-RAGE signaling pathway in diabetic complications	7.30E-05	Pathways of neurodegeneration - multiple diseases	0.003978292
Epstein-Barr virus infection	7.96E-05	Tryptophan metabolism	0.004229229
Toxoplasmosis	0.000113584	Herpes simplex virus 1 infection	0.00470776
Measles	0.000261619	Huntington disease	0.004904555
Viral myocarditis	0.000342298	Hepatitis C	0.005482644
Hepatitis B	0.000469354	Necroptosis	0.005679408
Influenza A	0.000576051	Legionellosis	0.007677517
Tuberculosis	0.000699058	Endometrial cancer	0.007940659
Kaposi sarcoma-associated herpesvirus infection	0.000926027	Steroid hormone biosynthesis	0.008754318
Amyotrophic lateral sclerosis	0.001219404		
