# Supplementary material for: Network pharmacology-based research on the effect of angelicin on osteosarcoma and the underlying mechanism
Source: Aging (Albany NY). 2023 Jun 10;15(11):5125–43. doi: 10.18632/aging.204786 (PMC10292874; doi:10.18632/aging.204786)
Supplement: Supplementary Table 3 [file aging-15-204786-s002.docx]

**Supplementary Table 3. The GO enrichment results for potential targets of angelicin in the treatment of osteosarcoma.**

| ONTOLOGY | Description | pvalue | ONTOLOGY | Description | pvalue |
| --- | --- | --- | --- | --- | --- |
| BP | response to drug | 2.03E-14 | BP | sex differentiation | 6.81E-06 |
| BP | xenobiotic transport | 5.74E-13 | BP | T cell homeostasis | 7.14E-06 |
| BP | response to metal ion | 1.17E-09 | BP | reactive oxygen species biosynthetic process | 7.24E-06 |
| BP | response to toxic substance | 2.90E-09 | BP | response to xenobiotic stimulus | 7.70E-06 |
| BP | bile acid and bile salt transport | 1.72E-08 | BP | intrinsic apoptotic signaling pathway | 8.08E-06 |
| BP | response to lipopolysaccharide | 2.15E-08 | BP | response to dexamethasone | 9.01E-06 |
| BP | response to molecule of bacterial origin | 3.33E-08 | BP | response to UV | 1.03E-05 |
| BP | female gonad development | 4.15E-08 | BP | response to estradiol | 1.06E-05 |
| BP | development of primary female sexual characteristics | 5.30E-08 | BP | response to ionizing radiation | 1.15E-05 |
| BP | response to copper ion | 8.84E-08 | BP | aging | 1.28E-05 |
| BP | female sex differentiation | 9.94E-08 | BP | response to tumor necrosis factor | 1.30E-05 |
| BP | drug transport | 1.03E-07 | BP | regulation of neuron death | 1.32E-05 |
| BP | drug metabolic process | 1.17E-07 | BP | steroid metabolic process | 1.55E-05 |
| BP | response to antibiotic | 1.27E-07 | BP | icosanoid transport | 1.65E-05 |
| BP | reactive oxygen species metabolic process | 2.76E-07 | BP | fatty acid derivative transport | 1.65E-05 |
| BP | ovarian follicle development | 2.81E-07 | BP | response to steroid hormone | 1.90E-05 |
| BP | secondary metabolic process | 2.81E-07 | BP | response to hypoxia | 2.27E-05 |
| BP | response to glucocorticoid | 2.86E-07 | BP | neuron death | 2.30E-05 |
| BP | export across plasma membrane | 3.95E-07 | BP | hydrogen peroxide metabolic process | 2.45E-05 |
| BP | response to corticosteroid | 4.92E-07 | BP | carboxylic acid biosynthetic process | 2.52E-05 |
| BP | hydrogen peroxide biosynthetic process | 5.84E-07 | BP | organic acid biosynthetic process | 2.55E-05 |
| BP | monocarboxylic acid transport | 6.60E-07 | BP | response to decreased oxygen levels | 2.65E-05 |
| BP | signal transduction in absence of ligand | 8.53E-07 | BP | positive regulation of neuron apoptotic process | 2.72E-05 |
| BP | extrinsic apoptotic signaling pathway in absence of ligand | 8.53E-07 | BP | cellular response to glucocorticoid stimulus | 2.72E-05 |
| BP | carboxylic acid transport | 8.64E-07 | BP | tetrapyrrole metabolic process | 3.00E-05 |
| BP | organic acid transport | 9.08E-07 | BP | lymphocyte homeostasis | 3.15E-05 |
| BP | lipid transport | 1.70E-06 | BP | regulation of B cell proliferation | 3.15E-05 |
| BP | regulation of neuron apoptotic process | 1.83E-06 | BP | anion homeostasis | 3.15E-05 |
| BP | gonad development | 2.24E-06 | BP | cellular response to corticosteroid stimulus | 3.15E-05 |
| BP | B cell proliferation | 2.40E-06 | BP | positive regulation of intrinsic apoptotic signaling pathway | 3.15E-05 |
| BP | cellular response to ketone | 2.40E-06 | BP | response to oxygen levels | 3.62E-05 |
| BP | development of primary sexual characteristics | 2.55E-06 | BP | steroid biosynthetic process | 3.95E-05 |
| BP | extrinsic apoptotic signaling pathway | 2.61E-06 | BP | response to ketone | 4.19E-05 |
| BP | B cell homeostasis | 3.11E-06 | BP | regulation of reactive oxygen species metabolic process | 4.19E-05 |
| BP | lipid localization | 3.26E-06 | BP | response to cobalt ion | 4.30E-05 |
| BP | reproductive structure development | 3.39E-06 | BP | regulation of mitochondrial membrane potential | 5.37E-05 |
| BP | neuron apoptotic process | 3.55E-06 | BP | xenobiotic catabolic process | 6.30E-05 |
| BP | reproductive system development | 3.57E-06 | BP | response to radiation | 6.45E-05 |
| BP | cellular response to dexamethasone stimulus | 3.82E-06 | BP | multi-multicellular organism process | 6.75E-05 |
| BP | response to nutrient levels | 4.95E-06 | BP | response to axon injury | 6.77E-05 |
| BP | xenobiotic metabolic process | 5.24E-06 | BP | programmed cell death involved in cell development | 7.45E-05 |
| BP | cellular response to xenobiotic stimulus | 6.18E-06 | BP | response to alcohol | 7.72E-05 |
| BP | leukocyte homeostasis | 8.41E-05 | BP | response to purine-containing compound | 0.000426354 |
| BP | post-embryonic development | 8.41E-05 | BP | negative regulation of neuron apoptotic process | 0.000426354 |
| BP | T cell activation | 9.31E-05 | BP | sensory perception of sound | 0.000451947 |
| BP | extrinsic apoptotic signaling pathway via death domain receptors | 9.31E-05 | BP | cholesterol metabolic process | 0.000460693 |
| BP | monocarboxylic acid biosynthetic process | 9.37E-05 | BP | estrogen metabolic process | 0.000468107 |
| BP | toxin metabolic process | 0.000100112 | BP | myeloid cell homeostasis | 0.000469547 |
| BP | glial cell apoptotic process | 0.000100112 | BP | response to amphetamine | 0.000498008 |
| BP | regulation of developmental pigmentation | 0.000100112 | BP | response to iron ion | 0.000498008 |
| BP | regulation of bile acid metabolic process | 0.000100112 | BP | eye development | 0.000513993 |
| BP | homeostasis of number of cells | 0.00010933 | BP | alcohol metabolic process | 0.000519057 |
| BP | execution phase of apoptosis | 0.000113048 | BP | DNA catabolic process, endonucleolytic | 0.000528816 |
| BP | tissue homeostasis | 0.000117801 | BP | long-chain fatty acid biosynthetic process | 0.000528816 |
| BP | positive regulation of neuron death | 0.000120266 | BP | response to arsenic-containing substance | 0.000528816 |
| BP | prostaglandin transport | 0.000129513 | BP | visual system development | 0.000534463 |
| BP | organic hydroxy compound transport | 0.00013045 | BP | secondary alcohol metabolic process | 0.000544344 |
| BP | intrinsic apoptotic signaling pathway in response to DNA damage | 0.000143706 | BP | apoptotic nuclear changes | 0.000560529 |
| BP | lymphocyte proliferation | 0.000160845 | BP | cellular response to alkaloid | 0.000560529 |
| BP | leukocyte apoptotic process | 0.000165367 | BP | positive regulation of mitochondrial outer membrane permeabilization involved in apoptotic signaling pathway | 0.000560529 |
| BP | mononuclear cell proliferation | 0.00016749 | BP | male sex differentiation | 0.000564165 |
| BP | epoxygenase P450 pathway | 0.000180612 | BP | sensory system development | 0.000566252 |
| BP | cellular response to tumor necrosis factor | 0.000193569 | BP | regulation of intrinsic apoptotic signaling pathway | 0.000584444 |
| BP | platelet formation | 0.000199504 | BP | cellular response to peptide | 0.000588181 |
| BP | cell aging | 0.000214831 | BP | sterol metabolic process | 0.00061573 |
| BP | platelet morphogenesis | 0.000219323 | BP | bile acid biosynthetic process | 0.000626663 |
| BP | leukocyte proliferation | 0.00023659 | BP | cellular component disassembly involved in execution phase of apoptosis | 0.000626663 |
| BP | icosanoid metabolic process | 0.000237024 | BP | sensory perception of mechanical stimulus | 0.000659088 |
| BP | response to ethanol | 0.000237024 | BP | cellular response to cadmium ion | 0.000661081 |
| BP | response to light stimulus | 0.000254374 | BP | regulation of apoptotic signaling pathway | 0.000675842 |
| BP | endoplasmic reticulum calcium ion homeostasis | 0.000261733 | BP | fatty acid derivative metabolic process | 0.000681481 |
| BP | B cell activation | 0.000282833 | BP | porphyrin-containing compound metabolic process | 0.000696397 |
| BP | regulation of steroid metabolic process | 0.000292269 | BP | regulation of inflammatory response | 0.000752385 |
| BP | apoptotic DNA fragmentation | 0.00030783 | BP | positive regulation of apoptotic signaling pathway | 0.000775909 |
| BP | response to immobilization stress | 0.00030783 | BP | regulation of lipid metabolic process | 0.000792884 |
| BP | transepithelial transport | 0.00030783 | BP | regulation of G1/S transition of mitotic cell cycle | 0.000800751 |
| BP | response to organophosphorus | 0.000312378 | BP | DNA catabolic process | 0.000846611 |
| BP | modified amino acid transport | 0.000332257 | BP | intrinsic apoptotic signaling pathway in response to oxidative stress | 0.000846611 |
| BP | detoxification | 0.000340552 | BP | vascular process in circulatory system | 0.000865065 |
| BP | amine metabolic process | 0.000355233 | BP | gland development | 0.000878497 |
| BP | cellular response to copper ion | 0.000357601 | BP | cellular response to metal ion | 0.000905187 |
| BP | male gonad development | 0.000362725 | BP | response to peptide hormone | 0.000908437 |
| BP | development of primary male sexual characteristics | 0.000370318 | BP | response to nicotine | 0.000927065 |
| BP | homeostasis of number of cells within a tissue | 0.000383859 | BP | protein insertion into mitochondrial membrane | 0.000927065 |
| BP | response to hydrogen peroxide | 0.000401714 | BP | regulation of mitochondrial outer membrane permeabilization involved in apoptotic signaling pathway | 0.000927065 |
| BP | protein insertion into mitochondrial membrane involved in apoptotic signaling pathway | 0.000411031 | BP | cellular response to amyloid-beta | 0.000927065 |
| BP | regulation of B cell activation | 0.000932583 | BP | necrotic cell death | 0.001866447 |
| BP | female pregnancy | 0.000946477 | BP | positive regulation of mitochondrial membrane permeability | 0.001924545 |
| BP | response to glucose | 0.000960502 | BP | protein insertion into membrane | 0.001924545 |
| BP | developmental pigmentation | 0.000968623 | BP | regulation of cholesterol metabolic process | 0.001924545 |
| BP | thymus development | 0.000968623 | BP | cellular response to extracellular stimulus | 0.001969989 |
| BP | regulation of small molecule metabolic process | 0.00097844 | BP | regulation of calcium ion transport | 0.001969989 |
| BP | response to oxidative stress | 0.0009945 | BP | regulation of mitochondrial membrane permeability involved in apoptotic process | 0.001983498 |
| BP | response to amine | 0.001011066 | BP | positive regulation of membrane permeability | 0.002043303 |
| BP | retinol metabolic process | 0.001011066 | BP | organic hydroxy compound biosynthetic process | 0.002082958 |
| BP | response to hexose | 0.001032612 | BP | regulation of multicellular organism growth | 0.002103959 |
| BP | bile acid metabolic process | 0.001054392 | BP | intracellular receptor signaling pathway | 0.002247809 |
| BP | anatomical structure homeostasis | 0.001086189 | BP | amyloid precursor protein metabolic process | 0.00229102 |
| BP | cellular response to steroid hormone stimulus | 0.001092712 | BP | sensory organ morphogenesis | 0.002345563 |
| BP | regulation of cell cycle G1/S phase transition | 0.001092712 | BP | demethylation | 0.002355066 |
| BP | establishment of protein localization to mitochondrial membrane | 0.001098601 | BP | T cell differentiation in thymus | 0.002619665 |
| BP | response to monosaccharide | 0.001108076 | BP | regulation of mitochondrial membrane permeability | 0.002687911 |
| BP | cellular response to lipopolysaccharide | 0.001123577 | BP | kidney development | 0.002708464 |
| BP | programmed necrotic cell death | 0.001143689 | BP | response to insulin | 0.002708464 |
| BP | divalent inorganic cation transport | 0.001165532 | BP | intrinsic apoptotic signaling pathway by p53 class mediator | 0.002756993 |
| BP | negative regulation of neuron death | 0.001186955 | BP | G1/S transition of mitotic cell cycle | 0.002818182 |
| BP | cellular response to interleukin-6 | 0.001189657 | BP | renal system development | 0.002959163 |
| BP | regulation of cysteine-type endopeptidase activity involved in apoptotic process | 0.001235946 | BP | cellular response to UV | 0.002969234 |
| BP | regulation of lymphocyte proliferation | 0.001303236 | BP | response to osmotic stress | 0.003263824 |
| BP | cellular response to nutrient levels | 0.001337732 | BP | activation of cysteine-type endopeptidase activity involved in apoptotic process | 0.003339532 |
| BP | regulation of mononuclear cell proliferation | 0.001337732 | BP | regulation of membrane permeability | 0.003493412 |
| BP | cellular response to inorganic substance | 0.001337732 | BP | cell cycle G1/S phase transition | 0.003502627 |
| BP | cellular response to molecule of bacterial origin | 0.001355195 | BP | primary alcohol metabolic process | 0.003974639 |
| BP | response to ischemia | 0.001382279 | BP | response to cAMP | 0.003974639 |
| BP | response to interleukin-6 | 0.001382279 | BP | cellular response to external stimulus | 0.004034128 |
| BP | mitochondrial outer membrane permeabilization | 0.001382279 | BP | urogenital system development | 0.004174289 |
| BP | response to gamma radiation | 0.001432615 | BP | cellular response to abiotic stimulus | 0.004209791 |
| BP | response to amyloid-beta | 0.001432615 | BP | cellular response to environmental stimulus | 0.004209791 |
| BP | arachidonic acid metabolic process | 0.001535892 | BP | cell death in response to oxidative stress | 0.004226195 |
| BP | response to carbohydrate | 0.001556863 | BP | camera-type eye development | 0.004245477 |
| BP | negative regulation of apoptotic signaling pathway | 0.001556863 | BP | regulation of steroid biosynthetic process | 0.004311658 |
| BP | response to reactive oxygen species | 0.001595444 | BP | pigmentation | 0.004397923 |
| BP | release of cytochrome c from mitochondria | 0.001642632 | BP | positive regulation of secretion | 0.004537663 |
| BP | regulation of cysteine-type endopeptidase activity | 0.001674403 | BP | regulation of calcium ion transport into cytosol | 0.004572857 |
| BP | regulation of leukocyte proliferation | 0.00169452 | BP | fatty acid transport | 0.004572857 |
| BP | positive regulation of mitochondrial membrane permeability involved in apoptotic process | 0.001697296 | BP | cholesterol homeostasis | 0.004572857 |
| BP | regulation of tumor necrosis factor-mediated signaling pathway | 0.001752821 | BP | sterol homeostasis | 0.004661522 |
| BP | intrinsic apoptotic signaling pathway in response to endoplasmic reticulum stress | 0.001809205 | BP | response to alkaloid | 0.00484124 |
| BP | mitochondrial outer membrane permeabilization involved in programmed cell death | 0.001809205 | BP | response to virus | 0.004880738 |
| BP | cellular response to biotic stimulus | 0.001818419 | BP | positive regulation of reactive oxygen species metabolic process | 0.005024134 |
| BP | response to cadmium ion | 0.001866447 | BP | positive regulation of protein transport | 0.005077981 |
| BP | negative regulation of calcium ion transport | 0.001866447 | BP | negative regulation of extrinsic apoptotic signaling pathway | 0.005116767 |
| BP | retinoid metabolic process | 0.00521019 | BP | retina development in camera-type eye | 0.009826808 |
| BP | limbic system development | 0.0053044 | BP | small molecule catabolic process | 0.009950373 |
| BP | cellular response to chemical stress | 0.005321002 | BP | positive regulation of cysteine-type endopeptidase activity | 0.009952761 |
| BP | lymphocyte differentiation | 0.005655866 | CC | apical plasma membrane | 1.96E-05 |
| BP | long-chain fatty acid metabolic process | 0.005689088 | CC | apical part of cell | 4.68E-05 |
| BP | positive regulation of establishment of protein localization | 0.005741532 | CC | pore complex | 0.000223455 |
| BP | diterpenoid metabolic process | 0.005787214 | CC | brush border membrane | 0.001430683 |
| BP | response to amino acid | 0.005787214 | CC | membrane raft | 0.003741807 |
| BP | unsaturated fatty acid metabolic process | 0.006187489 | CC | membrane microdomain | 0.00377382 |
| BP | positive regulation of mitochondrion organization | 0.006289493 | CC | membrane region | 0.004205414 |
| BP | olefinic compound metabolic process | 0.006495813 | CC | brush border | 0.004684738 |
| BP | terpenoid metabolic process | 0.006811051 | MF | xenobiotic transmembrane transporter activity | 1.50E-13 |
| BP | gland morphogenesis | 0.006811051 | MF | ATPase-coupled transmembrane transporter activity | 4.75E-08 |
| BP | apoptotic mitochondrial changes | 0.00691766 | MF | primary active transmembrane transporter activity | 6.68E-08 |
| BP | fatty acid metabolic process | 0.00692725 | MF | active transmembrane transporter activity | 9.17E-07 |
| BP | regulation of metal ion transport | 0.00692725 | MF | ATPase-coupled ion transmembrane transporter activity | 2.80E-05 |
| BP | negative regulation of G1/S transition of mitotic cell cycle | 0.007025031 | MF | cysteine-type endopeptidase activity involved in apoptotic signaling pathway | 4.55E-05 |
| BP | platelet degranulation | 0.007351703 | MF | BH domain binding | 5.55E-05 |
| BP | cellular response to light stimulus | 0.007351703 | MF | death domain binding | 5.55E-05 |
| BP | vitamin metabolic process | 0.007573269 | MF | ATPase activity | 5.66E-05 |
| BP | cellular amine metabolic process | 0.007573269 | MF | organic anion transmembrane transporter activity | 8.45E-05 |
| BP | response to acid chemical | 0.007685183 | MF | efflux transmembrane transporter activity | 9.17E-05 |
| BP | negative regulation of cell cycle G1/S phase transition | 0.007685183 | MF | cysteine-type endopeptidase activity involved in apoptotic process | 0.000105775 |
| BP | signal transduction in response to DNA damage | 0.007797849 | MF | chaperone binding | 0.000160369 |
| BP | cellular hormone metabolic process | 0.007911265 | MF | bile acid transmembrane transporter activity | 0.000171838 |
| BP | positive regulation of cysteine-type endopeptidase activity involved in apoptotic process | 0.007911265 | MF | modified amino acid transmembrane transporter activity | 0.000190813 |
| BP | cellular response to glucose stimulus | 0.007911265 | MF | heat shock protein binding | 0.000289201 |
| BP | digestive tract development | 0.00802543 | MF | aromatase activity | 0.000300355 |
| BP | B cell receptor signaling pathway | 0.00802543 | MF | lipid transporter activity | 0.000338577 |
| BP | cellular response to hexose stimulus | 0.008140343 | MF | anion transmembrane transporter activity | 0.000433048 |
| BP | B cell differentiation | 0.008256002 | MF | ion transmembrane transporter activity, phosphorylative mechanism | 0.000526022 |
| BP | cellular response to monosaccharide stimulus | 0.008256002 | MF | carboxylic acid transmembrane transporter activity | 0.000589703 |
| BP | calcium ion transport | 0.008465523 | MF | oxidoreductase activity, acting on paired donors, with incorporation or reduction of molecular oxygen, reduced flavin or flavoprotein as one donor, and incorporation of one atom of oxygen | 0.00059204 |
| BP | establishment of protein localization to mitochondrion | 0.008607441 | MF | organic acid transmembrane transporter activity | 0.000600367 |
| BP | mitochondrial membrane organization | 0.008845438 | MF | steroid hydroxylase activity | 0.000698209 |
| BP | cell junction assembly | 0.00890722 | MF | demethylase activity | 0.000698209 |
| BP | regulation of endopeptidase activity | 0.00890722 | MF | Hsp70 protein binding | 0.000853024 |
| BP | regulation of tube diameter | 0.008965543 | MF | Hsp90 protein binding | 0.000853024 |
| BP | regulation of blood vessel diameter | 0.008965543 | MF | organic hydroxy compound transmembrane transporter activity | 0.001113422 |
| BP | isoprenoid metabolic process | 0.009086385 | MF | nuclear receptor activity | 0.001305639 |
| BP | regulation of tube size | 0.009086385 | MF | ligand-activated transcription factor activity | 0.001305639 |
| BP | protein localization to mitochondrion | 0.009086385 | MF | active ion transmembrane transporter activity | 0.001603014 |
| BP | regulation of peptidyl-serine phosphorylation | 0.009207961 | MF | monocarboxylic acid transmembrane transporter activity | 0.001677471 |
| BP | cellular response to carbohydrate stimulus | 0.009207961 | MF | protein heterodimerization activity | 0.004173116 |
| BP | regulation of membrane potential | 0.009420216 | MF | monooxygenase activity | 0.004824582 |
| BP | digestive system development | 0.00945331 | MF | cysteine-type endopeptidase activity | 0.006104461 |
| BP | regulation of mitotic cell cycle phase transition | 0.009712621 | MF | protease binding | 0.008705244 |
| BP | regulation of protein-containing complex assembly | 0.009771739 | MF | heme binding | 0.008827835 |
| BP | multicellular organism growth | 0.009826808 | MF | cysteine-type peptidase activity | 0.008951205 |
